# Mapping Disease Data: A Usability Test of an Internet-Based System of Disease Status Disclosure

**DOI:** 10.3389/fvets.2017.00230

**Published:** 2018-01-05

**Authors:** Gareth Enticott, Andrew Mitchell, William Wint, Nigel Tait

**Affiliations:** ^1^School of Geography and Planning, Cardiff University, Cardiff, United Kingdom; ^2^Animal and Plant Health Agency, Addlestone, United Kingdom; ^3^ERGO, University of Oxford, Oxford, United Kingdom

**Keywords:** bovine tuberculosis, biosecurity, disease mapping, communication, risk based trading, usability research, veterinary epidemiology

## Abstract

Disease maps are important tools in the management of disease. By communicating risk, disease maps can help raise awareness of disease and encourage farmers and veterinarians to employ best practice to eliminate the spread of disease. However, despite the importance of disease maps in communicating risk and the existence of various online disease maps, there are few studies that explicitly examine their usability. Where disease maps are complicated to use, it seems that they are unlikely to be used effectively. The paper outlines an attempt to create an open access, online, searchable map of incidents of bovine tuberculosis in England and Wales, and analyzes its usability among veterinarians. The paper describes the process of creating the map before describing the results of a series of usability trials. Results show the map to score highly on different measures of usability. However, the trials also revealed a number of social and technical limitations and challenges facing the use of online disease maps, including reputational dangers, role confusion, data accuracy, and data representation. The paper considers the challenges facing disease maps and their potential role in designing new methodologies to evaluate the effectiveness of disease prevention initiatives.

## Introduction

Disease maps are important tools in the management of disease. On the one hand, disease mapping is used to detect relationships between human and animal diseases (1), disease incidence and prevalence, and social factors (2). On the other hand, the publication of animal disease maps represents what cartographers refer to as the “map communication model” of risk communication (3, 4). As well facilitating the management of animal disease by government veterinarians and policy makers (5), these maps can help raise awareness and vigilance among farmers and veterinarians (6). This communicative role of disease maps is increasingly important in the context of efforts by governments to reduce regulation and promote behavioral change. However, publicly available maps on disease risks are rare.^1^ Moreover, the map communication model has been criticized across a range of social policy domains for failing to deliver substantive changes in behavior (7, 8) where the apparent objectivity of maps is undermined by day-to-day experiences (9, 10).

Despite the apparent importance of using maps to communicate animal disease risks, there are few attempts to examine their use. Where disease maps have been created, it seems that they are complicated to use, require extensive training and only used by those responsible for data entry (11, 12). The aim of this paper is therefore to explore the usability of disease maps designed to improve the disease knowledge of veterinarians and farmers. Specifically, the paper examines the usability of a publically searchable online map of bovine tuberculosis (bTB) incidents—known as “Information Bovine Tuberculosis” (ibTB)—in England and Wales. While bTB is seen as the most serious animal disease threat in these countries (13), a common critique of government bTB policy has been the absence of information given to veterinarians and farmers about bTB incidents in their local area (14–16). In response to these criticisms, legislation was amended in October 2014 allowing the government to “publish information regarding that herd in any form that the Secretary of State sees fit for the purpose of helping other persons to protect against the further spread of tuberculosis” [(17): Article 10(14)].^2^ This coincided with the Open Government initiative (18)—a commitment to make government data available to the public to help generate better environmental and agricultural policy (19).

The paper examines the development and usability of the ibTB disease map through a series of usability trials with veterinarians. In doing so, the paper also explores the social and technical limitations and challenges facing the use of online disease maps to enhance and encourage greater levels of biosecurity on cattle farms.

## Materials and Methods

### Developing an Online Map of bTB Incidents

In order to make bTB incident data available, a searchable website was designed.^3^ The website (www.ibtb.co.uk) hosted data of all “ongoing” and “closed” bTB incidents since 2010 in England and Wales. Farms without a bTB history are not visible. The only data associated with individual incidents are their start and end date. Other information such as farm type and herd size is not displayed. The website is hosted in the cloud using MS Azure technology. It is a “3 tier application”—comprising data, service, and application tiers. To create the data tier ibTB utilizes data from the Animal and Plant Health Agency’s (APHA) “Sam” system which records bTB testing details. Data from this system are downloaded on a monthly basis, cleaned, re-formatted, and geo-referenced and then uploaded to the ibTB back-end SQL Server database. Other data utilized by the system are as follows: Ordnance survey’s Codepoint—used for the postcode search facility and the base maps (topographic, satellite, etc.) which are freely available ESRI products. Technologies utilized in the service and application layers included ASP.Net, HTML, C#, JavaScript, and ESRI JavaScript API. ESRI code was used to cluster breakdowns and display them as one of three categories (1 breakdown, 2–200, >200).

### Usability Testing

A series of usability trials were conducted to assess the ease of use of ibTB and users’ perceptions of the role ibTB could play in managing bTB. Usability is defined in ISO 9241-11 as comprising three key dimensions: satisfaction (i.e., users’ subjective reactions), efficiency (i.e., the level of resource consumed in performing tasks), and effectiveness (i.e., the ability and quality of users to complete tasks) (20, 21). A common approach to evaluating usability is to set users tasks and analyze how each is completed, measuring the number of mistakes and time taken [see, e.g., Ref. (22–24)]. Qualitative data on usability can be collected using a “thinking aloud protocol” in which narrate their actions while completing set tasks (25, 26). Quantitative data can be collected using survey methods [see, e.g., Ref. (27–29)]. Of these, Brooke’s (30) system usability survey (SUS) is widely recognized as the leading method (31). The SUS is a well-established 10-item survey used to examine “usability” and “learnability” (21). It has been cited in over 1,200 studies, incorporated into commercial usability toolkits and recognized as the industry standard (32). The SUS provides an overall level of usability but cannot act as a diagnostic (30), hence the need to combine qualitative and quantitative methods.

To assess the usability of ibTB, a task-based approach was adopted in which users were asked to complete five different tasks reflecting a range of activities that ibTB could be used for, varying in difficulty and geography (see Table 1). Users were asked to complete the tasks and using the “thinking aloud protocol” (26), commentate on their actions. Some usability studies use researchers as “chauffeurs” guiding users through tasks (25). In this case, researchers only intervened when asked direct questions. Researchers provided limited assistance when users did not know the location of a specific town or place (such as in Task 3), but did not intervene when users attempted unexpected workarounds (see “Results” section). After completing all tasks, participants completed a short survey containing the 10 SUS questions (see Table 2), and rate the ease of completion for each task along a 1–7 scale (1—very difficult, 7—very easy). A further four questions relating to participants’ use of online maps (1—not at all, 5—very often) and computing experience were asked (1—none, 5—high). Finally, participants were asked a series of open questions about how ibTB useful was for their work and the barriers and limitations to using it.

**Table 1:** Tasks used for user testing of ibTB.

Task	Description	Function	Geographical scale of search
1	Find out the bTB status of the last farm you visited? If they are under-restrictions, when did it happen?	General knowledge	Local
2	A farmer is worried because he has heard in the pub that one of his neighbors has gone down with bTB. Is the farmer right?	Checking neighbors	Local
3	One of your clients asks you about some cattle he’s interested in near XXXXX. What can you tell the farmer about the bTB situation around XXXXX? How many farms are currently under-restriction, and how many came off Tb restrictions in 2014?	Informed buying	Regional
4	A client is thinking of renting some ground near CPHH XXXX, but he doesn’t know the TB situation. What can you find out for the farmer? Are there any ongoing breakdowns in the area?	Advising farmers	Regional
5	You are writing a paper on bTB in the Low risk Area. How many ongoing bTB breakdowns are there in Norfolk, and how many farms had their restrictions lifted in 2014?	Epidemiology advocacy	National

**Table 2:** Results of the SUS survey (30).

Survey item	Dimension of usability (21)	Type of veterinarian (mean score)		
		APHA	Private sector	All veterinarians
I think I would like to use this system frequently	Usability	1.67	2.94	2.48
I found the system unnecessarily complex	Usability	3.22	3.63	3.48
I thought the system was easy to use	Usability	2.89	3.44	3.24
I think that I would need the support of a technical person to be able to use this system	Learnability	4.00	3.88	3.92
I found the various functions in this system were well integrated	Usability	2.67	3.00	2.88
I thought there was too much inconsistency in the system	Usability	2.78	2.94	2.88
I would imagine that most people would learn to use this system very quickly	Usability	3.44	3.31	3.36
I found the system very cumbersome to use	Usability	3.22	3.5	3.4
I felt very confident using the system	Usability	2.67	3.56	3.24
I needed to learn a lot of things before I could get going with this system	Learnability	3.11	3.88	3.6
SUS score		74.17	85.16	81.2

*All items are measured on a 1–5 (1––strongly disagree, 5––strongly agree)*.

*Calculation of the SUS score is achieved by converting the 1–5 scale to a 0–4 scale. For items 1, 3, 5, 7, and 9 the score contribution is the scale position minus 1. For items 2, 4, 6, 8, and 10, the contribution is 5 minus the scale position. The overall SUS value is calculated by multiplying the sum of the scores by 2.5 (30)*.

### Research Participants

Usability trials were conducted with farmers and veterinarians. This paper reports only on veterinarians’ views of ibTB. Veterinarians were included in usability testing because ibTB has the potential to provide veterinarians with a complete epidemiological picture of bTB in their area. As influential experts, veterinarians also play an important role in advising farmers on bTB (33, 34). Usability testing generally relies on between 5 (22) and 12 (31) participants. For this study, a total of 25 veterinarians were involved; nine from APHA, and 16 from private practices; located in high, low and medium bTB risk areas. Ethical consent was provided by Cardiff University’s Research Ethics Committee, and informed consent was obtained from participants before all usability testing. This involved explaining the nature of the research and providing assurances of confidentiality and anonymity.

### Data Capture and Analysis

Participants were provided with a laptop with external mouse to complete each task. Each participant’s activity was recorded using “Silverback”—a screen capture application that records screen activity, mouse clicks, and audio. Following each usability trial, the video was reviewed and instances of user frustration (drawing mouse circles and double clicking) were noted, along with the number of mistakes made during the task and the time taken to complete it. These were cross-checked with separate observation notes taken during each usability trial. On three occasions Silverback failed to record screen and/or audio activity. In a further two cases, there was no accessible WiFi or 3G signal available to connect to ibTB. Instead, the participant’s own computer was used to conduct the usability tests, but without the ability to record using Silverback. These users are excluded from the analysis of task completion. Responses to open-ended questions were recorded using a digital voice recorder. Transcripts were prepared and analyzed thematically to draw out shared uses, concerns and limitations relating to ibTB. Survey data were entered into SPSS to calculate the SUS scores, and conduct descriptive analysis and statistical tests of association and difference.

## Results

### User Characteristics

Veterinarians were asked about their use of maps and computers in their daily work. Use of computers was rated as very frequent (mean 4.92, 1–5 scale) and veterinarians generally responded that they felt comfortable using a computer (mean 4.44, 1–5 scale). Veterinarians’ use of maps (mean 3.92) and online maps (mean 3.68) was less frequent.

### User Views of Uses of ibTB

All veterinarians welcomed the development of ibTB. Private veterinarians in particular were pleased to be able to see these data, suggesting that the information was vital for them to work with their clients to help them manage bTB. These veterinarians argued that previous data restrictions reflected a perceived lack of trust in private veterinarians’ epidemiological skills by Government, but that ibTB could now help them engage with farmers and government veterinarians on an equal footing. In democratizing bTB information, veterinarians therefore saw three clear uses for ibTB:

(a) **Reassurance**: Veterinarians commented that ibTB could be used to show farmers that they are not the only ones with bTB in their area, thereby helping to reduce the stigma of a bTB incident on their farm. For example, one vet commented: “There’s a lot of stigma about TB in this area. It can be an issue. But if they see they’re not the only ones with it, that could help.”
(b) **Advising**: Veterinarians argued that farmers can frequently be unaware of the bTB situation in their local area. While farmers may claim to know who has bTB, often this knowledge is based on rumor. As one vet said: “Often, when you go out on a farm, they don’t know who else has got TB. Sometimes they say they do, but they’re wrong.” However, private veterinarians acknowledged that farmers can sometimes have a better knowledge of bTB than themselves. ibTB was seen as providing a valuable factual resource that effectively democratized information on bTB. As a result, some veterinarians argued that they were in a better position to provide advice to farmers on best practice such as biosecurity and cattle movements as each could know the “true” incidence of bTB in their local area.
(c) **Epidemiological knowledge**: Related to the second reason, private veterinarians argued that for too long they had been excluded from easy access to bTB data. Finding out the bTB status of a farm neighboring a client’s but registered to a different veterinary practice involved convoluted conversations with other veterinary practices in which client confidentiality meant that disclosing disease status was often problematic. ibTB therefore avoided these problems, but could also allow veterinarians to build up a picture of bTB in their local area and understand its spread.

Veterinarians also commented on the potential use of ibTB among farmers. While opinions varied on the extent to which farmers would use ibTB and the impact it would have on their behavior, the following three main uses were identified:

(a) **Nosiness**: Reflecting academic studies of farmer behavior (35) veterinarians argued that farmers are inherently interested in what goes on around them, particularly in relation to bTB. While rumors and conversations at pubs and markets could be the source of farmers’ knowledge, ibTB would provide a more accurate way of checking up on other farmers’ disease status.
(b) **Explaining tests**: The regulations around bTB testing can be complicated for farmers. Veterinarians argued that often farmers can be confused about why they have to test their herd so soon after their last herd test (this can be due to a new bTB incident on a nearby farm). Veterinarians reasoned that farmers could use ibTB to check local bTB to help explain any unexpected tests.
(c) **Farm biosecurity**: Some veterinarians suggested that farmers might use ibTB to identify whether they should improve their on-farm biosecurity (because of a nearby bTB incident) or assess the riskiness of potential stock purchases from an area or specific farm. However, many veterinarians were skeptical about the extent to which farmers would use ibTB to do this, suggesting that farmers’ purchasing habits were difficult to change. As one vet pointed out: “Most stock decisions we can’t change: traders will always trade; closed herds want to stay closed; there’s not many in the middle.”

### Task Completion

Results of the usability trials are shown in Table 3. In only four cases, tasks were not completed by users. In all cases, failure to complete the task was a result of not being able to accurately identify the farm searched for. When a user searches for a farm using its unique identification code (known as the CPHH), the map zooms in and centers over the farm. However, unless the farm has an ongoing outbreak, ibTB does not display a placeholder to indicate the farm location. For some participants, this meant they felt that were unable to complete the task. Others instantly assumed that this was the case and completed the task based on the assumption that the farm was “more or less by there,” or stated that they knew where the farm was located because of their local knowledge.

**Table 3:** Completion of tasks set in ibTB.

	Completion rate	Mean completion time (s)	Mouse circles (*n*)	Double clicks (*n*)	Mistakes (*n*)	Ease of completion (1–7 scale)
Task 1	19/20	121.55	2	6	12	5.88
Task 2	18/20	101.65	2	4	4	4.76
Task 3	20/20	159.25	3	11	11	4.92
Task 4	19/20	103.05	3	0	0	5.76
Task 5	20/20	144.65	9	8	1	5.32
All Tasks	96/100	126.03	19	29	28	5.32

*All users (*n* = 25) completed ease of completion questions. When Silverback failed to record or users activity could not be recorded (*n* = 5) are excluded from other results*.

The average time to complete a task was 126 s. Task 2 was completed quickest on average, while Task 3 had the slowest average although was completed by all participants. It is worth pointing out that variation in the time taken to complete the tasks was also due to the users’ level of interest in bTB. For example, some users were keen to explore in detail the incidence of bTB in surrounding herds for some tasks (e.g., Tasks 3 and 4) in order to form epidemiological judgments.

There were few visible signs of frustration among participants. Mouse circles were counted on 19 occasions, and double clicking on 29. One sign of frustration not recorded but which became evident during testing was respondents rapidly zooming in and out of the map. Sometimes this was due to poor mouse control but it also occurred when users were trying to find a specific location (such as during Tasks 3 and 5). One problem with the use of ESRI maps was that place names could disappear at different magnification levels making navigation awkward.

Mistakes were made by 17/20 users, although only a total of 28 individual errors were recorded across all five tasks. Twelve mistakes were recorded for Task 1. This could have been due to the fact that this was the first task. A common mistake was the incorrect use of the search function. On being asked to search for a farm using its CPHH, veterinarians frequently left out the final part of the code, resulting in a failed search. Task 3 also had a significant number of mistakes due to inappropriate searches. On being asked to search for a specific place, 10 users attempted to search by typing the place name into the search box. When this failed to work, users completed a Google search to find a postcode with which to search. The remainder navigated to the location where they thought the town was located before finding it. These mistakes highlighted the importance of including a place name search function in future versions of ibTB.

When asked to rate the ease of completing each task, all responses were positive. The average ease of completion for the five tasks was rated at 5.3 out of 7. Task 1 was rated easiest despite having most mistakes. Tasks 2 and 3 were rated lowest. In both cases, users experienced problems of identifying which farm they had searched for because of a failure of ibTB to show a placeholder for all farms. Users who rated the tasks as easy were also more likely to complete the tasks in less time (*r* = −0.514, *p* = 0.021). Users who rated themselves as more frequent computer users also completed tasks quicker than less frequent users (*r* = −0.586, *p* = 0.007). However, there was no relationship between experience with maps and time or ease of task completion.

### Usability of ibTB

Overall, ibTB scored highly on the System Usability Scale (see Table 2). The average usability score for all users was 81.2 (minimum = 55, maximum = 100). Users who rated ibTB highly also tended to rate the tasks as easy to complete (*r* = 0.457, *p* = 0.022) but the relationship between the SUS scores and time taken to complete tasks was not significant (*r* = −0.437, *p* = 0.054). There was also no relationship between SUS scores and users’ declared proficiency in using a computer or online maps. When different dimensions of usability are considered separately, users who completed the tasks quickly were more likely to rate ibTB as highly learnable (*r* = 0.541, *p* = 0.014). All other relationships were not statistically significant.

Items on the SUS to which users agreed most included the ease of use, the ease of learning how to use ibTB, and users’ confidence while using ibTB. Items on the SUS that received most negative feedback included the belief that the system was very cumbersome, inconsistent, and unnecessarily complex. This may reflect some of the mistakes and problems highlighted in “Task completion” section, such as the inability to search for places and not highlighting farms searched for.

### Differences between Users

Usability was rated highest among private veterinarians whose average score was 85/100. Government veterinarians working for APHA scored slightly lower (74/100), but both scores indicated above average usability. The lower scores for government veterinarians are likely to be due to the fact that other bTB resources are available to them. While private veterinarians were generally enthused by the availability of “new” data, veterinarians working in APHA commented that other mapping and data management software was more suitable.

### Social and Technical Limitations of ibTB

While the results of the SUS suggest ibTB to be highly usable, comments by veterinarians during and after the tasks revealed a number of social and technical limitations to the system. These are described as follows:

(a) **Reputational dangers**: Most veterinarians accepted that ibTB could function as a means to improve biosecurity and cattle purchasing decisions. However, not only were they skeptical about the extent to which farmers would change their behavior as a result of ibTB, but they also raised concerns about their role in passing on information about bTB status. For private veterinarians, the idea that they could now pass on previously confidential data about another farmers’ bTB status was troubling. Some veterinarians commented that they would be reluctant to tell farmers about others’ bTB status. Instead, they said that they would tell them to go and look on ibTB themselves, or direct them to an administrator in their practice who could look up the information for them. These actions were due to two concerns related to avoiding reputational damage to the practice or their career. The first was the need to avoid giving out inaccurate data that could threaten veterinarians’ business. Given that ibTB has a time lag of one month, this was seen as a real possibility. The second was a concern to be seen as an “informant.” One vet referred to this as a “TB Grass,” commenting that “The last thing I want is a reputation for going round telling people that so and so has TB.”
(b) **Role confusion**: While private veterinarians were concerned about their reputation, APHA veterinarians were concerned about how ibTB changed their role in bTB management. Because of the confidential nature of disease status, government veterinarians have been trained not to reveal the bTB status of neighboring farms to other farmers. That this information was now publicly available challenged this role, and veterinarians were unsure what it would mean for them in future. During the tasks, APHA veterinarians asked if they were allowed to tell farmers what ibTB said about farmers’ neighbors. Just like private veterinarians’ coping strategy, these veterinarians also suggested they would simply tell farmers to go and look on ibTB for themselves. In the minds of both private and public veterinarians, ibTB challenges their role as a neutral arbiter or a “bTB confidant” that they believed contributed to forging a trusting relationship with farmers. For policy makers, the lesson is that the social context of disease management affects how information on disease status is interpreted and distributed.
(c) **Accuracy**: The accuracy of data was a widespread concern. It was frequently stated by veterinarians that while the tasks were easy to complete, their accuracy was open to question. As noted above, one concern was the ability to identify a farm without a placeholder on the map. Although veterinarians could use local knowledge or assume their location, it remained a concern because as one vet described “it matters because I would give different advice depending on which one it is.” More broadly, veterinarians complained that the maps in ibTB did not show field boundaries which were important in attempting to make an epidemiological assessment of the riskiness of a farm they had searched for: “It doesn’t tell you where the breakdown occurred—it could have been miles away. That could affect your opinion on whether it was risky.” Others also pointed out that this assessment should also take into account the precise location of the breakdown given that herds are split into epidemiological groups that can be located in different parts of a farm. As one vet commented: “I wouldn’t rely on it too heavily for factual accuracy—for me, you don’t want to get it wrong in public—it isn’t quite good enough for that.”
(d) **Data absences**: In relation to data accuracy, veterinarians also pointed to other forms of data that ibTB could display that would also assist in making an informed epidemiological risk assessment for any given farm. These included the need to display all farms whether they had ever had bTB or not. This was mentioned often in relation to Task 5 where only a handful of cases were evident, but veterinarians wanted to get a sense of disease prevalence as well as incidence. Related to this was the absence of county boundaries on the map (these were only displayed at high magnification). While veterinarians accepted that disease “knew no boundary,” these could still be useful in getting a sense of prevalence in areas that were not familiar to veterinarians. Other data requested by veterinarians included: herd size; herd type; the number of reactors and inconclusive reactors in a breakdown; and the number, interpretation and type of tests used. Private veterinarians were also keen to learn the cause of the breakdown held in APHA records, and some argued that information from wildlife surveys and the *M. bovis* genotype should be shown on the map. The purpose of showing these data was to help inform their own epidemiological assessment of a farm and/or area, but some veterinarians also suggested it could be important in demonstrating to the public the need for wildlife controls to prevent the spread of bTB.
(e) **Data representation**: Many veterinarians commented that some aspects of the visual display could be improved to help interpret the maps better. Important place names could disappear at different levels of magnification. The clustering of breakdowns was also criticized. First, the 3-point scale was seen to be ineffective, and second, clusters could move when uses zoomed in and out of the map. This had the effect of changing users’ assessment of the level of bTB in an area. The zoom function could also change users’ perceptions of an area in other ways. When searching for a CPHH (e.g., in Task 4), the default zoom level could portray a different disease picture compared with a wider zoom level. It was noticeable that among those veterinarians that took the time to zoom out there was often a change in their perception of the incidence of bTB in the area searched for, once they realized that there were many other breakdowns nearby. Finally, some veterinarians commented that it would be better to see how many years each farm had been free from bTB. This was perceived to be more positive than current breakdowns which as one vet suggested could look like “a map of doom.” Years of freedom is also familiar to farmers as a metric used in risk based trading mechanisms for bTB and other diseases (36).

## Discussion

Despite the significance of maps and mapping to epidemiology and disease management, it is surprising to find few studies of their usability in animal disease management. The usability trials conducted for this research have proved useful in helping to further refine ibTB to enhance its usefulness. This has included the following: making changes to the appearance of the map; including a placeholder to identify the location of each search; changing clustering breakdowns by dynamically creating nested grids depending on the map resolution, counting the breakdowns in each cell and clustering if > 20; expanding the range of clusters to 5 (1 breakdown, 2–20, 21–150, 151–500, >500); and making changes to the search facility. The website has also been made fully compatible with mobile devices. Technical limitations have meant that the ability to search for place names—one of the main problems faced by users—has not been included.

The usability trials demonstrated that veterinarians had high levels of satisfaction, could complete tasks easily, and rated ibTB highly on the SUS. However, despite the high levels of usability, veterinarians still identified key technical and social challenges that affected their use of ibTB. For some, these challenges suggest the need for further research on the way maps represent and communicate disease data. The usability trials suggested the need for a broader consideration of how different representations of bTB can influence farmer and vet behavior. Studies on behavior change argue that the way a phenomenon is framed can impact upon subsequent behavior. For example, research suggests that avoiding losses is often a greater behavioral driver than winning (37). Framing bTB data in a loss aversion frame—such as by emphasizing the number of years free of disease—may act as an aspirational target and incentive to protect (i.e., avoid loss) of their disease-free rating. Moreover, avoiding a negative framing in which disease incidents are emphasized may avoid reinforcing farmers’ and veterinarians’ feelings that “there is nothing they can do” (38). There is currently a range of different attempts to rank bTB risk, framed in different ways (36, 39). The extent to which these spatial representations of disease risk enhance requires further research.

On the other hand, thinking about how disease maps represent data should also direct disease mappers to consider the underlying assumptions and power relations within their maps. Veterinarians’ responses to ibTB revealed tensions within the assumption that they should play an important in using and promoting disease maps to farmers. For example, while previous research has highlighted the importance of veterinarians in encouraging farmers to implement biosecurity or advise them in animal health (34), discussions with veterinarians revealed tensions between the aims of “responsibility sharing” and perceptions of their role in encouraging responsible conduct among farmers. For some veterinarians, the need to protect one’s career, business, and reputation meant distancing themselves from their role in transmitting open data, delegating that responsibility to other colleagues or farmers themselves. Despite their important role in advising farmers on animal health, these results suggest that their role cannot be assumed and that as veterinarians balance competing interests, their role may play out differently. This may lead to varying degrees of support for new technologies, such as disease maps, designed to change farmer behavior and establish new norms of appropriate conduct.

Similarly, the usability trials of ibTB also highlighted the contested boundaries of who or what counts as epidemiology—what critical cartographers refer to as the underlying political choices and meanings hidden within maps (40). These critical perspectives on mapping encourage us to examine the politics of representation and the actual use of maps in practice (3). In this case, private veterinarians recalled how they had been distanced from official bTB data, acting as data collectors, but not data analysts: a division not just of labor, but also of epidemiological status. However, the development of technologies like ibTB can act as devices to re-engage different epidemiological divisions, potentially resulting in new approaches to disease management (41). While ibTB shows that disease maps can disrupt tradition patterns of veterinary practice, it also shows how these shifts are dependent on decisions over which data to make public. Further research should be directed at understanding the role of animal disease mapping across all diseases in shaping the boundaries between veterinary and epidemiological disciplines, and their effect upon the management of disease. This should include an examination of the very assumptions behind why disease maps are used and for whom. In the case of ibTB, it was assumed that farmers and veterinarians would use the maps to manage disease but without a registration system, this is difficult to verify. In fact, a range of unexpected users have challenged these assumptions, using ibTB to protest against the use of wildlife controls (i.e., badger culls) to control bTB. The announcement of new badger cull zones has been accompanied by spikes in ibTB use that are linked to anti-badger culling activists using ibTB to fact-check claims made by government, veterinary and farming representatives about bTB incidence, and to evaluate the effectiveness of badger culls on bTB incidence (see, e.g., https://web.archive.org/web/20150910011759/http://badger-killers.co.uk/culling-increases-btb/). While farmers’ groups have called for access to ibTB to be strictly controlled (42), their response again highlights the extent to which disease maps inscribe the boundaries of epidemiology and their power to limit who can speak about animal disease.

## Conclusion

Despite the importance of maps in animal disease epidemiology, there have been few studies analyzing their effectiveness of communicating animal disease risks to farmers, veterinarians, and the public. This paper describes the use of animal disease surveillance data for bTB to create a freely available, searchable online map of current and historic incidents of bTB in England and Wales. In usability tests with veterinarians, the paper shows that epidemiological tasks could be completed quickly and easily, and usability was rated excellent. In doing so, the paper shows the potential role for disease maps in evaluating farmer behavior and devising effective mechanisms for risk-based trading. However, the paper also shows that the creation of disease maps can challenge traditional sets of power relations in disease management, question what counts as epidemiology, and require choices to be made over who can and cannot participate in the management of disease. As open data initiatives become more common as governments seek innovative digital solutions to intractable problems, disease mappers will need to find ways of resolving these challenges in order for disease maps to make effective contributions to the management of animal disease.

## Ethics Statement

This study was carried in accordance with the recommendations from the Research Ethics Committee at the School of Geography and Planning, Cardiff University. All research participants provided their consent following an information briefing on the nature of the research. Participants have been anonymized in the research findings.

## Author Contributions

AM, WW, and NT designed and created the ibTB website. GE designed, conducted, and analyzed results from the usability trial. GE wrote the manuscript which was agreed by all authors.

## Conflict of Interest Statement

ibTB was produced with funding from the Department for Environment, Food and Rural Affairs (Defra). AM is employed by the Animal and Plant Health Agency (APHA), an agency of Defra. Interpretation of the results of the usability trial rests with the authors. The reviewer DD and handling Editor declared their shared affiliation.

## Footnotes

^1^Attempts to make maps of disease outbreaks available on-line include the OIE’s World Animal Health Information Database (WAHIS) (http://www.oie.int/wahis_2/public/wahid.php/Wahidhome/Home). Other examples of online maps of animal disease include: http://www.healthmap.org; Refsral knowledge http://diseasemaps.usgs.gov/mapviewer/; http://www.wormsandgermsmap.com; https://www.capcvet.org/parasite-prevalence-maps.

^2^Animal disease policy is a responsibility for the devolved administrations in Wales, Scotland and Northern Ireland. Wales altered legislation in 2015, whilst Scotland and Northern Ireland have chosen not to make this information available.

^3^The website was commissioned by the Department for Environment, Food and Rural Affairs (Defra) who are responsible for bTB policy in England.
